# 8-Year Changes in Frailty in Adults: Links to Cognitive and Physical Function and Mortality

**DOI:** 10.1093/geroni/igab046.2190

**Published:** 2021-12-17

**Authors:** Felicia Simpson, Jamie Justice, Judy Bahnson, Joni Evans, Kathleen Hayden, Stephen Krtichevsky, Karen Johnson, Mark Espeland

**Affiliations:** 1 Department of Mathematics, Winston-Salem State University, Winston-Salem, North Carolina, United States; 2 Wake Forest School of Medicine, Wake Forest School of Medicine, North Carolina, United States; 3 Wake Forest School of Medicine, Winston-Salem, North Carolina, United States; 4 University of Tennessee Health Science Center, Memphis, Tennessee, United States

## Abstract

Deficit accumulation frailty indices are being evaluated as clinical markers of biological aging. In this context, it is to be expected that changes over time in such indices should be predictive of downstream changes in cognition, physical function, and mortality. We derived a frailty index (FI) based on deficit accumulation in 38 functional, behavioral, and clinical characteristics and examined associations between 8-year changes in FI and subsequent standardized measures of cognitive and physical function and mortality collected over years 8-18. We drew data from the Look AHEAD clinical trial of a multidomain intensive lifestyle intervention (ILI) in 3841 adults, aged 45-76 years at baseline with overweight/obesity and type 2 diabetes mellitus. Greater FI increases tended to occur among individuals who were older, non-Hispanic White, heavier, and who had greater baseline multimorbidity. Greater increases in FI were associated with subsequently worse levels of composite cognitive function and 400m walk speed (all p<0.001). Additionally, compared with the lowest tertile of FI change, hazard ratios [95% confidence intervals] for 10-year mortality for the middle and highest tertiles of FI change were 1.28 [1.03.1.58] and 1.56 [1.24,1.96], respectively. While assignment to ILI was associated with smaller 8-year increases in FI, this did not translate overall to better cognitive functioning compared to the Diabetes Support and Education control condition across years 8-18. Increase in FI over 8 years predicts subsequent reduced function and greater mortality. However, whether interventions generally targeting FI reduce risks for downstream outcomes remains to be seen.

